# Factors affecting aseptic loosening of 4750 total hip arthroplasties: multivariate survival analysis

**DOI:** 10.1186/1471-2474-8-69

**Published:** 2007-07-24

**Authors:** Barbara Bordini, Susanna Stea, Manuela De Clerico, Sergio Strazzari, Antonio Sasdelli, Aldo Toni

**Affiliations:** 1 Laboratorio di Tecnologia Medica, Istituti Ortopedici Rizzoli Via Barbiano 1/10, 40136 Bologna, Italy; 2 Servizio di Farmacia, Istituti Ortopedici Rizzoli Via Barbiano 1/10, 40136 Bologna, Italy; 3 Direzione Amministrativa, Istituti Ortopedici Rizzoli Via Barbiano 1/10, 40136 Bologna, Italy; 4 I Divisione di Ortopedia e Traumatologia, Istituti Ortopedici Rizzoli Via Barbiano 1/10, 40136 Bologna, Italy

## Abstract

**Background:**

Total hip arthroplasty is a successful surgery, that fails at a rate of approximately 10% at ten years from surgery. Causes for failure are mainly aseptic loosening of one or both components partially due to wear of articular surfaces and partially to design. The present analysis aimed to identify risk factors and quantify their effects on aseptic failure.

**Methods:**

Multivariate survival analysis was applied to 4,750 primary total hip arthroplasties performed between 1995 and 2000.

**Results:**

The survival of the prosthesis is affected by gender, age, pathology, type of the prosthesis and skill of the. The worst conditions are male patients, younger than 40 years, affected by sequelae of congenital diseases, operated by a who performed less than 400 total hip artroplasty in the period. Furthermore, cemented cups and stems (less expensive) have a higher risk of failure compared with uncemented ones (more expensive).

**Conclusion:**

The only variable that affects survival and that can be modified by is the type of prosthesis: a lower cost is associated to a higher risk. Results concerning the risk associated with cemented components are partially in disagreement with studies performed in countries where cemented prostheses are used more often than uncemented ones.

## Background

From a surgical point of view, total hip arthroplasty is a well-standardized operation that has proven to be very effective. However, failure can sometimes occur in the immediate postoperative period or even some years after. According to the NICE guidelines, for a hip prosthesis to be considered safe its mean survival rate should be at least 90% at ten years [1]. Although the failure rate is low, it varies greatly and can be influenced by several factors, such as the type of prosthesis used and the patient's characteristics, and whereas the patient's characteristics are practically unchangeable, factors related to s and their choices can be modified. The literature includes numerous studies analysing factors that influence hip prosthesis failure, but they often concern small series of patients. Poon observed that weight and age influenced the outcome of total hip arthroplasty (THA) using cemented prostheses [2]. Kobayashi studied a consecutive series of 293 primary cemented Charnley prostheses and found that rapid wear of polyethylene and abnormal geometry of the femoral medullary canal affect prosthesis survival [3]. Kolundzic found that demographic factors explained only a minor part of the survival variability of 82 cementless acetabular cups [4].

The largest series concerned only patients treated using cemented prostheses. Among them Berry analysed 2000 primary Charnley prostheses at 25 years' follow-up and found that age, gender and underlying diagnosis affected long-term survivorship of both components [5]. Dawson compared 598 cemented prostheses and found no significant differences between the performance of the two models [6]. As clearly stated the weakness of all the long-term studies depends on the fact that they assess the success or failure of old technologies and designs [7]. Comparison among prostheses with different fixation (cementless vs cemented) is limited to few clinical trials that indicate the better performance of cementless components. [8-10].

Analyses performed on data from northern European registers only partially fill this gap since there is a clear-cut prevalence of cemented prostheses with metal-polyethylene couplings.

In fact, 93.1% of prostheses implanted in patients in Sweden between 1979–2004 [11], and 80% of those used in Norway between 1987–2004 [12] were cemented. The figure is lower in Denmark, 49.8% between 1995–2004 [13], and in Finland 55% from 1980–2003 [14]. The data collected for the UK were only for 2004, and although they included cemented prostheses in 49% of cases, they cannot be used for an effective analysis [15].

However, an analysis of the data from the Norwegian resister Furnes revealed that in over 53,000 operations some diseases (femoral neck fracture sequelae, congenital hip dysplasia, and rare diseases) represent risk factors for prosthesis survival [16].

When limiting the analysis to young osteoarthritic patients, Eskelinen found that age and gender influence the result [17].

The Danish register identified age and gender as confounding factors in the evaluation of prosthesis survival [13].

None of these analyses, due to the nature of the operations analyzed, considered the influence of the type of prosthesis fixation among the possible risk factors. Only when analysing the Finish data Visuri find that low age, male gender, uncemented prostheses and first 10 year-period of surgery were risk factors for loosening of the prostheses [18]. Therefore, we analyzed the data of a series of patients with a minimum of six years' follow-up taken from the RIPO register (Register of Orthopedic Prosthetic Implantology), which includes cemented and uncemented prostheses, to determine the influence of patient characteristics, 's experience, and type of prosthesis used, on the outcome of the operation.

## Methods

### Materials

A consecutive series of 4,750 primary total hip arthroplasties performed on 4,450 patients at Istituto Rizzoli between 1^st ^January 1995 and 31^st ^December 2000 was analysed. The number of operations progressively increased from 1995 (664) to 2000 (847). The patients were treated in 11 different wards. They underwent regular clinical evaluation, and if they missed their clinical examination for longer than 18 months they were contacted by phone, to establish whether the prosthesis was still in place. The survival of the prosthesis was recorded at the time of death.

The characteristics of patients are shown in Table 1 and in Table 2.

**Table 1:** Characteristics of the studied group. Patients are classified according variables that entered in subsequent multivariate analysis

	**Type of cup**		
	**Cemented all polyethylene**	**Press fit, polyethylene liner**	**Press fit, ceramic or metal liner**

	N. (%)	N. (%)	N. (%)
**Gender**			
Male	137 (24.3)	765 (39.5)	897 (39.9)
Female	426 (75.7)	1173 (60.5)	1352(60.1)
**Side**			
Right	303 (53.8)	1052 (54.3)	1191 (53.0)
Left	260 (46.2)	886 (45.7)	1058 (47.0)
**Age at surgery**			
Lower than 40	2 (0.4)	80 (4.1)	326 (14.5)
Between 40 and 69	151 (26.8)	1311 (67.7)	1503 (66.8)
Higher than 70	410 (72.8)	547 (28.2)	420 (18.7)
**Charnley's hip score**			
Class A	360 (63.9)	1294 (66.8)	1373 (61.0)
Class B+C	203 (36.1)	644 (33.2)	876 (39.0)
**Skills of surgeon**			
Less than 400 operations (68 surgeons)	352 (62.5)	1225 (63.2)	569 (25.3)
More than 400 operations (4 surgeons)	211 (37.5)	713 (36.8)	1680 (74.7)
**Diagnosis in primary arthroplasty**			
Primary arthritis	299 (53.1)	1156 (59.6)	1212 (53.9)
Sequelae of congenital and pediatric diseases	35 (6.2)	321 (16.6)	509 (22.6)
Femoral neck fracture and sequelae	158 (28.1)	272 (14.0)	251 (11.2)
Other	71 (12.6)	189 (9.8)	277 (12.3)

**Table 2:** Characteristics of the studied group. Patients are classified according variables that entered in subsequent multivariate analysis

	**Type of stem**				
	**Straight cemented**	**Anatomic cemented or straight cemented with particular design**	**Straight cementless**	**Surface treated or anatomic cementless**	**Modular cementless**

	N. (%)	N. (%)	N. (%)	N. (%)	N. (%)
**Gender**					
Male	482 (32.7)	167 (39.5)	300 (38.4)	414 (37.6)	436 (45.0)
Female	994 (67.3)	256 (60.5)	482 (61.6)	687 (62.4)	532 (55.0)
**Side**					
Right	829 (56.2)	227 (53.7)	398 (50.9)	588 (53.4)	504 (52.1)
Left	647 (43.8)	196 (46.3)	384 (49.1)	513 (46.6)	464 (47.9)
**Age at surgery**					
Lower than 40	25 (1.7)	5 (1.2)	159 (20.3)	92 (8.4)	127 (13.1)
Between 40 and 69	748 (50.7)	256 (60.5)	574 (73.4)	663 (60.2)	724 (74.8)
Higher than 70	703 (47.6)	162 (38.3)	49 (6.3)	346 (31.4)	117 (12.1)
**Charnley's hip score**					
Class A	937 (63.5)	304 (71.9)	511 (65.3)	670 (60.9)	605 (62.5)
Class B+C	539 (36.5)	119 (28.1)	271 (34.7)	431 (39.1)	363 (37.5)
**Skills of surgeon**					
Less than 400 operations (68 surgeons)	710 (48.1)	393 (92.9)	538 (68.8)	378 (34.3)	127 (13.1)
More than 400 operations (4 surgeons)	766 (51.9)	30 (7.1)	244 (31.2)	723 (65.7)	841 (86.9)
**Diagnosis in primary arthroplasty**					
Primary arthritis	831 (56.3)	296 (70.0)	321 (41.1)	676 (61.4)	543 (56.1)
Sequelae of congenital and pediatric diseases	155 (10.5)	28 (6.6)	278 (35.5)	171 (15.5)	233 (24.1)
Femoral neck fracture and sequelae	300 (20.3)	70 (16.5)	81 (10.4)	141 (12.8)	89 (9.2)
Other	190 (12.9)	29 (6.9)	102 (13.0)	113 (10.3)	103 (10.6)

They received cemented (12.1%), cementless (51.5%), or hybrid (36.4%) total hip prostheses.

Femoral and acetabular components were classified on the basis of their characteristics and economic value. Relative value was calculated respectively as a ratio to the value of all-polyethylene cup encompassing the cost of cement and to the value of a straight cemented stem. In Table 3 and 4 cups and stem are classified respectively into three and five groups where the main characteristics and relevant economic values are reported. Details of the different types of cup/stem that compose each group are also presented.

**Table 3:** Distribution of implanted cups, according to fixation and type of liner

*Cup*	*Relative value*	*n. implants*
**Cemented all polyethylene**	1.00	563
135 Contemporary Howmedica (Mahwah, New Jersey, USA)		
149 Muller Cremascoli (Milano, Italy),		
130 Muller Sulzer (Geneve, Switzerland),		
54 Exeter Howmedica,		
other types with less than 50 implants each.		

**Press fit, polyethylene liner**	3.73	1938
375 Duraloc De Puy (Warsaw, Indiana, USA)		
343 Fitek Sulzer,		
336 Duofit PSF Samo (Bologna, Italy),		
250 ABG Howmedica,		
123 Vitalock Talon Howmedica,		
105 PCA Howmedica,		
other types with less than 100 implants each.		

**Press fit, ceramic or metal liner**	4.94	2249
1548 AnCA Fit Cremascoli,		
276 Duofit PSF Samo,		
144 Fitek Sulzer,		
133 Standard Cup Protek		
other types with less than 100 implants each		

**Total**		4750

**Table 4:** Distribution of implanted stems, according to fixation and design

*Stem*	*Relative value*	*n. implants*
**Straight cemented stem**	1.00	1476
473 LC Samo,		
344 AHS Cremascoli,		
198 Definition Howmedica,		
169 Exeter Howmedica,		
103 Gemini De Puy,		
70 self-locking Sulzer,		
other types with less than 50 implants each		

Anatomic cemented or straight cemented with particular design	1.16	423
228 Elite De Puy,		
191 Lubinus SP 2 Link,		
other types with less than 100 implants each.		

**Straight cementless**	1.88	782
502 Cone prosthesis Sulzer,		
120 Meridian Howmedica,		
85 CLS Sulzer,		
62 Metabloc Sulzer,		
other types each with less than 50 implants.		

**Surface treated or anatomic cementless**	2.10	1101
567 Anca Cremascoli,		
194 Duofit RKT Samo,		
125 PCA Howmedica,		
98 Citation Howmedica,		
77 AML Depuy stems		
other types each with less than 50 implants		

**Modular cementless**	2.46	968
869 Anca Fit Cremascoli,		
86 Dual fit Cremascoli stems,		
other types each with less than 10 implants		

**Total**		4750

The s were classified on the basis of their experience, i.e. the number of operations performed as primary in the five-year period. All the surgeons implanted the hip prosthesis by lateral approach.

### Statistics

Implant survivorship was estimated with use of the Cox proportional -hazards model [19]. Ninety – five percent confidence intervals were calculated. The death of a patient or or the revision of any component was recorded. All cases that failed due to septic loosening were excluded. The end point for the acetabular component was revision of the metal back and/or of the liner. The end-point for the femoral component was revision of the stem and/or of the modular neck (if present). Revision of the neck due to head damage was not considered considered as modular neck failure; in those cases the neck is revised as a precautionary measure, and is not an index of stem failure. For patients that suffered a cup failure their follow-up time would not be registered at the date of this failure when analysing stem failures and viceversa. It was preliminarly verified that variables entering the model were not different among patients suffering for a single component failure or for contemporary failure of both stem and cup.

Variables included gender, age, diagnosis, Charnley score, right or left side, surgeon's skill, type of component. Cox multivariate test enables verification of the influence of one variable on equal terms with the others.

## Results

### The results are presented separately for the two main components, cup and stem

The chi-square test used to test globally the model applied was significant if, on the whole, the variables put into the model influenced significantly the outcome of prosthetic surgery (chi-square for cup = 52.49; chi-square for stem = 69.604, both significant AT *p *= 0.001). In the analysis of cup failure the total number of valid observations was 4,750, of which 4,616 were not removed and 134 were revised (Table 5). 46 patients out of 134 had cup and stem failure at the same time.

**Table 5:** Results of multivariate analysis of cup failure

	*Failures/I Implants*	*Unadjusted (95% CI)*	Adjusted for all variables *(95% CI)*	*P*
***Gender***				
Female	*82/2951*	*1 (referent)*	*1(referent)*	
Male	*52/1799*	*1.12 (0.79–1.58)*	*0.76 (0.53–1.10)*	*p = 0.16*
***Side***				
Left	*60/2204*	*1 (referent)*	*1 (referent)*	
Right	*74/2546*	*1.08 (0.77–1.52)*	*1.16(0.82–1.63)*	*p = 0.34*
***Age at surgery***				*P *_--*trend*=_*0.005*
Between 40 and 69	*86/2965*	*1 (referent)*	*1 (referent)*	
Higher than 70	*27/1377*	*0.75 (0.49–1.16)*	*0.66 (0.41–1.08)*	*p = 0.098*
Lower than 40	*21/408*	*1.99 (1.24–3.21)*	*2.02 (1.21–3.38)*	*p = 0.007*
***Charnley's hip score***				
Class A	*78/3027*	*1 (referent)*	*1(referent)*	
Class B+C	*56/1723*	*1.12 (0.79–1.57)*	*1.15 (0.81–1.63)*	*p = 0.42*
***Skills of surgeon***				
More than 400 operations	63/2604	*1 (referent)*	*1 (referent)*	
Less than 400 operations	71/2146	*1.46 (1.04–2.05)*	*1.23 (0.85–1.77)*	*p = 0.27*
***Type of cup***				*P *_--*trend*=_*0.004*
Press fit, ceramic or metal liner	50/2249	*1 (referent)*	*1 (referent)*	
Cemented all polyethylene	23/563	*1.84 (1.12–3.01)*	*2.68 (1.48–4.83)*	*p = 0.001*
Press fit, polyethylene liner	61/1938	*1.42 (0.98–2.06)*	*1.53 (1.02–2.29)*	*p = 0.04*
***Diagnosis in primary arthroplasty***				*P *_--*trend*=_*0.0001*
Primary arthritis	51/2667	*1 (referent)*	*1 (referent)*	
Sequelae of congenital and pediatric diseases	44/865	2.44 (1.63–3.65)	2.32 (1.49–3.62)	p = 0.0001
Femoral neck fracture and sequelae	29/681	2.41 (1.53–3.80)	1.98 (1.24–3.17)	p = 0.004
Other	10/537	0.99 (0.50–1.95)	0.46 (0.38–1.55)	p = 0.46

The outcome is not significantly affected by clinical condition, right or left side, or surgeon's skill. In the analysis of stem failure the total number of valid observations was 4,750, of which 4,645 were not removed and 86 were revised.

Outcome is not significantly affected by Charnley score, side, or diagnosis.(Table 6) Both cup and stem survival are negatively affected by age under forty, and cemented fixation of the components. Besides this, the cup survival is also negatively affected by a preoperative congenital disease or fracture and sequelae. On the contrary, stem survival is not affected by the pathology, but is negatively affected by male gender and lower surgeon skill.

**Table 6:** Results of multivariate analysis of stem failure

	*Failures/Implants*	*Unadjusted*	Adjusted for all variables *(95% CI)*	*P*
***Gender***				
Female	*39/2951*	*1 (referent)*	*1 (referent)*	
Male	*47/1799*	2.09 (1.37–3.19)	2.00 (1.28–3.12)	*p = 0.002*
***Side***				
Left	*38/2204*	*1 (referent)*	*1 (referent)*	
Right	*48/2546*	1.10 (0.72–1.69)	1.21 (0.78–1.85)	p = 0.39
***Age at surgery***				*P *_--*trend*=_*0.048*
Between 40 and 69	*58/2965*	*1 (referent)*	*1 (referent)*	
Higher than 70	*18/1377*	*0.73 (0.43–1.24)*	0.58 (0.33–1.00)	*p = *0.18
Lower than 40	*10/408*	1.37 (0.70–2.69)	*1.66 (0.81–3.44)*	*p = *0.02
***Charnley's hip score***				*P *_--*trend*= 0.120_
Class A	*45/3027*	*1 (referent)*	*1 (referent)*	
Class B+C	*41/1723*	1.46 (0.95–2.22)	*1.56 (1.02–2.39)*	*p = 0.40*
***Skills of surgeon***				
More than 400 operations	28/2604	*1 (referent)*	*1 (referent)*	
Less than 400 operations	58/2146	2.670 (1.70–4.19)	1.99 (1.21–3.29)	*p = *0.007
***Type of stem***				*P *_--*trend*= 0.001_
Straight cemented stem	41/1476	*1 (referent)*	*1 (referent)*	
Anatomic cemented or straight cemented with particular design	14/423	1.59 (0.86–2.95)	1.19 (0.63–2.26)	*p = 0.59*
Straight cementless	13/782	0.64 (0.34–1.19)	0.45 (0.23–0.88)	*p = 0.019*
Surface treated or anatomic cementless	8/1101	0.24 (0.11–0.51)	0.24 (0.11–0.52)	*p = 0.0001*
Modular cementless	*10/968*	0.45 (0.23–0.90)	0.46 (0.22–0.98)	*p = 0.045*
***Diagnosis in primary arthroplasty***				*P *_--*trend*=_*0.001*
Primary arthritis	43/2667	*1 (referent)*	*1 (referent)*	
Sequelae of congenital and paediatric diseases	12/865	0.80 (0.42–1.52)	0.99 (0.50–1.99)	*p = 0.99*
Femoral neck fracture and sequelae	23/681	2.26 (1.36–3.75)	1.84 (1.09–3.12)	*p = 0.02*
Other	8/537	0.94 (0.44–2.00)	0.75 (0.34–1.65)	*p = 0.48*

## Discussion

To determine the factors that influence component survival we followed a large number of consecutive primary total hip arthroplasties performed in the same Institute. The cohort was large enough to be analysed by age, gender, diagnosis, Charnley score, side, skill of surgeon, and type of component. Distribution of frequencies of some variables (ie age at surgery, surgeon experience) is clearly different among types of implants. Multivariate analysis applied to test the influence of single factors can limit this bias. By analysing prostheses implanted between 1995–2000, we were able to include designs that are still modern, and at the same time have a long enough follow-up to highlight any failures.

Since the register was started at Rizzoli Institute in 1990, all patients have been monitored; if patients fail to attend scheduled clinical exams, they are contacted by phone or asked to fill in a questionnaire. This was acceptable as the recorded end-point (revision) was independent of clinical examination. The chosen end-point is undoubtedly a raw parameter, which does not take into account the quality of life and restoration of function in the treated limb, but its strength lies precisely in its objectivity.

Some of the results obtained from this analysis support data reported by other authors in comparable series. In agreement with the literature, the risk of failure is increased by male gender, young age, and certain diseases [17,18,20]. These variables, which constitute the patient's characteristics, are unchangeable. However, knowing the influence they can have enables a correct statistical interpretation. The interesting finding that has emerged from this study is that among the factors that influence the risk of failure are the surgeon's skill, and the type of prosthesis-to-bone fixation used.

The surgeon's skill is an extremely delicate aspect, which might depend on the reliability of the hospital where the operation is performed rather than the experience of the single surgeon. High-risk patients, who are often admitted to hospitals not necessarily near home, might be treated more safely in highly specialised centres. It should be remembered that the data presented in this study come from operations performed at a highly specialised hospital and includes very complex cases, which, on the other hand, have been treated by highly specialised surgeons.

Another important factor that can be modified is the prosthetic component. Uncemented components are generally much less likely to fail than cemented ones. However, our results appear to be in contrast with those of other registers [11]. Nevertheless, reading the data more carefully reveals that as experience using uncemented components increases, the difference in results between the two types of prostheses decreases, and the efficacy of uncemented prostheses is highlighted especially with regards to young [12,17] or middle-aged patients [13].

An interesting finding that emerged from our study was that the more expensive the prosthesis, the longer its survival was.

With regards to the cup, all other variables being equal, compared to the monoblock polyethylene cup the failure rate of the press-fit cup with a polyethylene liner, which costs four times more than the monoblock cup, was reduced by half, and reduced by 2/3 when using the press-fit cup with a ceramic liner, which costs five times more than the monoblock cup.

Concerning the stem, there were no significant differences in the failure rate between the straight cemented stem and the anatomical cemented stem, which costs 10% more. Conversely, compared to the cemented straight stem, the failure rate of the uncemented straight stem, which costs 90% more than the cemented one, is 60% less. The reduction in the failure rate is 60% also when using uncemented modular stems, which cost 150% more than the cemented straight stem.

Finally, coated and/or anatomical uncemented stems cost 110% more than cemented straight stems but the failure rate is reduced by 80%.

All the conclusions drawn from these data have intrinsic and unavoidable limits due to the low rate of revision (less than 3%) that affect primary Total Hip Arthroplasty. The revision rate is fortunately lower than the 10% suggested as the maximum acceptable by NICE [1]. For this reason a non-parametric statistical method of analysis was used, which can handle correctly this kind of data.

This analysis provides the basis for a cost-benefit assessment, which aims at determining whether a certain clinical result can be achieved while reducing the resources used. From a strictly ethical point of view the results give a clear indication of the choice, but the availability of economic resources can only be determined by healthcare policy. Undoubtedly, subsequent cost-benefit analysis should take into account that this type of operation is performed on elderly people who need a long recovery period. Therefore, there is also a need for rehabilitation centres, which are often lacking, and so elderly people often have to rely on the help of their families.

Besides social aspects, also technical difficulties should not be underestimated. Sometimes surgeons are faced with difficult operations and have to make bold choices. However, cost-benefit analysis is not within the scope of this paper, which is limited to providing data to enable correct elaboration [21,22]. We reiterate that the data presented come from a series of patients and include the use of cemented and uncemented components, unlike those based on large databanks of northern European registers, which show that cemented components perform better [23,24] or at least as well as [25] uncemented ones. Since cemented prostheses are cheaper, they are more advantageous from a cost-benefit point of view. The data we have presented, which do not include only the cost of materials [26] will enable a cost-benefit analysis that is closer to reality in countries where the use of uncemented prostheses is more widespread.

## Conclusion

The only variable that affects survival and that can be modified by surgeon is the type of prosthesis: a lower cost is associated to a higher risk. Results concerning the risk associated with cemented components are partially in disagreement with studies performed in countries where cemented prostheses are used more often than uncemented ones.

## Competing interests

The author(s) declare that they have no competing interests.

## Authors' contributions

BB and MDC performed statistical analysis, SuS drafted the manuscript, AT gave its experience as Orthopedic Surgeon, SeS and AS collected and treated data on cost.

## Pre-publication history

The pre-publication history for this paper can be accessed here:


